# Supplementary material for: Perceptions and attitudes towards unmanned aerial vehicles (drones) use for delivery of HIV medication among fisher folk communities on the Islands of Kalangala, Uganda
Source: PLOS Glob Public Health. 2024 Aug 22;4(8):e0003468. doi: 10.1371/journal.pgph.0003468 (PMC11340983; doi:10.1371/journal.pgph.0003468)
Supplement: S1 Text — (PDF) [file pgph.0003468.s001.pdf]

FGD AND KEY INFORMANT INTERVIEW GUIDE

Study Title: Use of Unmanned Air Vehicles (Medical Drones) to overcome Geographical barriers to delivery Antiretroviral Therapy: The Bufumira Pilot.

Introduction:

Hi, my name is ______________________ and I want to thank you for joining us today. I am helping to coordinate this study on the use of unmanned air vehicles (Medical Drones to deliver ART to adult patients eligible/enrolled in community based differentiated service delivery models in Bufumira Islands, Kalangala District. We are conducting this research to identify your views about the feasibility of using drones for ART delivery and also determine the impact of patient outcomes.

I would like to say that there is no right or wrong answer in our conversation. We will simply be discussing your views, opinions and experiences on a range of topics, so please feel comfortable to say what you honestly feel. I would like to tape record the whole session. Please do not be concerned about this: all measures will be taken by the researchers to maintain confidentiality of the interviews. Information you tell us will ONLY be used for this research project. As we are tape recording the interview, we ask that you refrain from using names or identifying information of yourself or your partners. If at any time during the interview you feel uncomfortable you can ask for a break, refuse to answer any question, and are always free to leave. Do you have any questions before we start?

Makerere University School of Public Health • [illegible] and Ethics Committee
Valid Thru:
24 MAY 2021

Create rapport: How have you been?

Questions to guide the interview

1. How do PLHIV access treatment (ART) in this area? *Describe the ways through which ART treatment is accessed by or delivered to PLHIV.*

2. What are the challenges faced when **accessing ART**? (*Probe for Financial, Physical, Transport related challenges in case they are not mentioned etc.)*

3. What are the challenges faced during **delivery of ART**? (*Probe for Financial, Physical, Transport related challenges in case they are not mentioned etc.)*

4. Suggest ways how the above challenges you have mentioned can be addressed to improve:

   a) Delivery of ART and other HIV supplies and products to hard-to reach populations.

b) Accessing ART and other HIV supplies and products in hard-to reach areas.

5. What do you know about medical drones/air vehicles?

6. What do you think about using drones to deliver the following in this area/hard-to-reach areas? (*Probe for their perceptions)*
   a) ART to PLHIV?
   b) Other medical/use cases?

7. How best do you think drones can be used to deliver ART to patients in this area/hard-to reach areas? *(Feasibility)*

8. Would you be willing to get your drugs delivered to you by the drones? *(Perceived Acceptability)*
   a) If Yes, explain why.
   b) If No, explain.

9. Do you think that using drones will help to ease accessibility to ART in this area? Why?

10. How else can drones be used in delivering health services?

11. Explain the advantages of drone delivery compared to other ART service delivery models (e.g. individual care at health centre, outreach from health centre to landing sites, community by boat, community drug distribution by drone) in Bufumira Sub-County, Kalangala District.

12. Tell us about any problems you foresee with the drones.

Probes:

- Community related problems
- Health care worker related
- Security related, etc.

Makerere University School of Public Health • Higher Degrees, Research and Ethics Committee •
Valid Thru:
24 MAY 2021
