# Supplementary material for: Perceptions and attitudes towards unmanned aerial vehicles (drones) use for delivery of HIV medication among fisher folk communities on the Islands of Kalangala, Uganda
Source: PLOS Glob Public Health. 2024 Aug 22;4(8):e0003468. doi: 10.1371/journal.pgph.0003468 (PMC11340983; doi:10.1371/journal.pgph.0003468)
Supplement: Attachment — Submitted filename: Reviewer report.docx [file pgph.0003468.s002.docx]

**Reviewer Report**

**General comments and introductions**

This is a significant study in the context of providing Universal Health Coverage in Uganda. However, the manuscript is not ready for publication yet and needs some major work, as detailed below:

The paper requires a thorough review of grammar and other language-related issues. I recommend you consider an English language editor to go through your paper and improve its quality.

The scope of the introduction is limited. Link the introduction to Universal Health Coverage, UHC, and SDGs context. HIV continue to be a major global public concern; we need to see the linkages with existing frameworks.

In your introduction, provide context on HIV prevalence in Uganda and globally. This is missing.

In your introduction, you suggested that achieving 95:95:95 is challenging, especially in low-income countries, could you provide an example?

Please put acronyms in full for the first time i.e., UPHIA, UNAIDs

**Title**

The title appears too long. It could be made more sharper.

The authors should stick to either drone or Unmanned Aerial Vehicles rather than repetition in the title—suggestion: Perceptions and attitudes towards the usage of drones for HIV medication delivery in Kalangala, Uganda.

**Method section**

Authors should pay attention to the method and provide the details:

The paper has not answered the question of study design and sample.

The study setting does not provide enough relevant detail i.e., the population, sub-population of people living with HIV and number of health facilities.

Justify the choice of recruitment method and data collection approach (FGD & IDI)

How did authors review interview records to inform subsequent interviews? Were there no debriefing sessions to review interview records to obtain insight to aid further interviews?

Provide details on the composition of the FGDs with justification.

In the last paragraph of the data collection section, the statement, “Interviewers asked respondents a series of questions ranging from their knowledge of drones to if they had ever seen them and what they are used for” does suggest you deploy Liker scale, but cannot be found in the paper, if l am wrong then rephrase for clarity.

How were participants' data anonymised?

In the last statement of the first paragraph on data analysis, the authors indicated that the lead social scientist checked for consistency and accuracy. Provide further details of the process in line with data credibility.

**Theory**

The manuscript lacks a theoretical perspective. Considering the novelty of the study, l recommend the authors apply theory such as Technology Acceptance Model (TAM) to predict behaviour and provide a theoretical explanation for the study.

**Results**

The results are quite interesting, but it does not provide a clear storyline. It will be great to have the perspectives of the health workers, PLHIV, community members, and opinion leaders in a way that provides sequence. What the agreements and disagreements are?

**Discussion**

The discussion must be in tandem with the results and situated within the literature, as it stands it does not.

The first and second paragraphs of the discussion section be added to improve the introduction.

Authors should attach the research instruments.

**Conclusion**

From the results, it was clear that the use of drones for the delivery of ART is a piece of welcome news for all stakeholders. Still, PLHIV are mindful of two critical issues about drone delivery: The reduction/elimination of face-to-face contact with health professionals and the stigma associated with the delivery of only ART. This has the tendency to defeat the essence of the project i.e., provide access. These must be highlighted in the study for future projects to take into consideration. These key areas are missing in your conclusion and recommendations.
