# Supplementary material for: Perceptions and attitudes towards unmanned aerial vehicles (drones) use for delivery of HIV medication among fisher folk communities on the Islands of Kalangala, Uganda
Source: PLOS Glob Public Health. 2024 Aug 22;4(8):e0003468. doi: 10.1371/journal.pgph.0003468 (PMC11340983; doi:10.1371/journal.pgph.0003468)
Supplement: Attachment — Submitted filename: Response to Reviewers.doc [file pgph.0003468.s003.doc]

19^th^ Jan 2024

Managing Editor

Editor in Chief

PLOS Global Public Health

**Ref.: PGPH-D-23-01417**

Perceptions and attitudes towards unmanned aerial vehicles (drones) use for delivery of HIV medication among fisher folk communities on the Islands of Kalangala, Uganda

Dear Sir/Madam,

We thank the reviewers for their helpful comments, which have improved the paper. Please find below our point-by-point responses (in bold italics) and line number for the revised **text in the marked-up manuscript.**

**Reviewer #1**: Thank you for allowing me the opportunity to review this interesting manuscript, Perceptions and attitudes towards unmanned aerial vehicles (drones) use for delivery of HIV medication among fisher folk communities on the Islands of Kalangala, Uganda. The topic is timely and of significant societal importance. Adherence to HIV medications is at the backbone of ensuring the UNAIDS goals. Despite these strengths, there were a number of significant issues that I hope will help you strengthen this manuscript.

1). **This paper will require a thorough editorial review in terms of grammar, spelling, double wording, and sentence structure in places. Please make sure all acronyms are spelled out (i.e., UPHIA, page 6, WHO, etc),**

*Thanks for the advice, the whole manuscript has been checked, revised thoroughly and the grammatical errors corrected, and a clean manuscript has been submitted. The acronyms have all been spelt out. For example, FGD to Focus Group Discussion on line number 24*

2**). On top of page 6, there appears to be HIV cited, but with differing numbers.**

*We appreciate your review, this has been rectified. The numbers are first for general prevalence and then broken down to prevalence of adults 15 years and above.*

*“According to the Uganda Population-based HIV Impact Assessment (UPHIA) 2020, the overall prevalence of HIV/AIDS in the general population was estimated at 5.5%. For adults (15 years and above), the prevalence was estimated at 5.8% with higher prevalence in women (at 7.2%) than in men (4.3%). The adult Viral Load Suppression (VLS) was 75.4% which is below 95% target” Line number 71 to 75*

**3). Page 6, second para, which aim?**

*Thank you for this comment, this has been corrected to read*

*“To address these barriers, the World Health Organization (WHO) and governments (including Uganda) have endorsed differentiated service delivery (DSD) models for HIV care, a patient centered ART delivery approach” line number 89*

**4) In the literature review, there is an entire section on barriers to HIV adherence which appears to be missing. This will be foundational to your study.**

*Thank you so much. This has been added.*

*“Retention in HIV care and adherence to HIV medication is affected by both individual and health system level barriers including (but are not limited to) long distances with high cost of travel, long clinic waiting times, stigma and frequent ART stock-outs” line number 84 to 86*

**5) HIV adherence data is not presented in the background as well.**

*Thank you so much for your observation, we have added a section on factors that hinder retention which in turn affects adherence.*

*“Client retention in care is critical to improve adherence levels which is the cornerstone for achieving targeted viral suppression rates. Recent reports indicated low retention rates for clients initiated in HIV care, ranging from 65-75% against the UNAIDS 95% target….” Line number 78 to 84*

**6) Last paragraph on page 6, I believe there is inconsistent information on prevalence of HIV again.**

*We appreciate your observation, this has been corrected.*

*“…compared to national prevalence average of 5.5%”. line number 107.*

**7) No information on prevalence of drowning events mentioned on page 7**

*Thank you so much, this has been added*

*“Studies have shown that a total of 2,066 drowning cases were reported with 1,332 (64%) deaths in 14 districts around Lake Victoria” Line number 130 to 133*

**8) Under Methods, what is the type of qualitative approach used. There are different types which have nuanced steps in completing.**

*Thank you so much for your comment, this has been added as follows:*

## Study design

## *“This was an exploratory, cross-sectional, qualitative study using FGDs and IDIs as data collection methods”. line number 148*

**9) Page 8, how were participants selected? What approaches were used?**

*Thank you so much, this has been added under “Participants and recruitment” from line number 171 to 220*

**10) Page 9, any information about the sites in terms of demographics, prevalence of HIV, etc**

*We appreciate this comment. There is no information on HIV prevalence or demographics available per site.*

**11) What were inclusion and exclusion criteria?**

*Thank you, these have been mentioned under “Participants and recruitment”*

*“Participants that showed interest in both the FGDs and IDIs were screened as per inclusion and exclusion criteria before written informed consent was obtained. Those eligible had to be 18 years and above or emancipated minors (15 years to 18 years) who were receiving ART from Bufumira health centre III (HCIII) or were not living with HIV but received care from the Bufumira HCIII. They should have been a health care worker at Bufumiira HCIII, should have stayed in Bufumira sub county for at least 12 months and were willing to stay for another 12 months, were considered a key opinion leader and should have provided a signed consent form. Participants were excluded if they did not meet the above criteria, or give consent or were known by the community as having had a mental illness” line number 214 to 220*

**12) Page 9, first full paragraph, last sentence. There is a lack of clarity on how participants were channeled into either focus groups or individual interviews. Now clear who participated in which approach.**

*We thank you for your observation, this has been clarified from line 181 to 213*

**13) Page 10, how was mental illness assessed and considered as an exclusionary criteria?**

*Thank you so much, the wording has been corrected as it is in the protocol as below. Line number 220.*

*“…were known by the community as having a mental illness”*

**Who recruited the participants?**

*The social scientists together with the local leaders as seen below.*

*“To ensure representative data, the social science team (JS, JB,) purposively recruited participants for both FGDs and IDIs according to age, sex and location together with the local leaders.” Line number 173*

**14) Page 10, end of first para. Unclear how the semi structured interview guide was assembled and by whom? Were there suggestions by the community for types of questions to be asked?**

*Thank you so much. We value community engagement and we involved the community leaders from the initiation. That is how we were allowed to put the weather masts up. We have included how the interview guide was developed together with sample questions that were in the guide.*

*“All interviews were conducted face to face, in both English and Luganda languages using a semi-structured interview guide that was developed by the lead social scientist (JS.) The interview guide was developed with consideration of concerns and guide from the community leaders that were engaged at the study initiation phase.*

*We included open- ended questions like what they thought drones were, what they are used for, if they have ever seen them before, how they feel about the drones delivering their ART and what they recommend the means of delivery would be. Line number 234 to 241*

**15) Page 11, end of first two lines. Any processes for assuring trustworthiness and credibility of the data?**

*Thank you for your comment, the process has been included.*

*“Data was cross-checked and verified by lead social scientist (JS) for consistency and accuracy. Preliminary data analysis occurred concurrently with data collection. The codes from the two social scientists were discussed in three research team meetings which helped draw attention to emerging themes. This was an important part of the preliminary analysis and identification of thematic categories” line number 264 to 273*

**16) Page 12, Any data on sensitization as this may impact findings of the study as stated.**

*Thank you for your advice, before study initiation, we had to engage with the community. Through this, we mentioned medical drones that were going to deliver ART from the Health Center to the island settlements. We have added what we told them on line number 327 to 329*

*“They were told about the possibility of bringing medical drones to the islands to deliver ART”*

**17) Page 18, A bit more information in the introduction on existing use of drones and how they are received by the community.**

*Thank you so much, a sentence has been added. Line number 135 to 136.*

*“Despite the growing interest in medical drones, there is limited data on perceptions of patients on its use since projects such as this one had not previously existed.”*

***“*Reviewer #2: The paper has major methodological gap and lacks scientific rigor.**

*Thank you, we have reviewed the methods section line number 145 to 258*

**The discussions and conclusion are not consistent with the results.**

*We appreciate your observation, we have revised the discussion to be consistent with the results. From line number 505 to 513*

*“Our findings highlight the perceived benefits and risks of medical drone use among a variety of participants in a hard-to-reach region of Uganda. We found evidence of perceived benefits around many critical elements in health service delivery including, reduced workload for health care providers, timely access to medication, reduced cost in transport, improved adherence to medical appointments. These are key factors that address important health system barriers in this region of the country. The perceived risks that of potential for reduced contact with health workers if medical drones efficiently deliver ART, possible increased stigma if medical drones only deliver HIV drugs and a fear of keeping the medical drone safe were mentioned. These perceived risks are understandable and important but are not complicated to mitigate.”*

**The analysis has some deficiencies which in my view could be resolved by the authors.**

*Thank you so much, this has been corrected as seen from line number 259 to 287*

**The study is a qualitative one and may only require including the tools and indicating availability of data upon request. This has not been made clear though.**

*Thank you so much, we have clearly stated that data will be available from line 280 to 281*

*“All this data is available upon request to those ethically allowed to access it”.*

*We have attached our data collection tools in the submission.*

**The paper poorly written grammatically. Require major English language editing.**

*Thank you so much for advice. The entire manuscript has gone through grammatical changes review.*

**Reviewer #3**: **The authors present their research on a novel and promising approach to delivering HIV medications to people living with HIV in islands that are difficult to reach by conventional transportation methods. The research supports the need to study medical drones and provides valuable insight into the acceptability of their use in the Islands of Kalangala. Despite some grammatical and punctuation issues, the paper is largely clearly written and well-organized, with findings that are highly relevant to ongoing challenges in HIV medication delivery.**

*Thank you so much for your comment.*

General comments: Line numbers would be helpful for reviewing the manuscript. While errors were not substantial and did not get in the way of writers’ clear communication, the manuscript could benefit from some additional copyediting to address grammatical and punctuation issues.

***Thank you so much for your advice, line numbers have been added and the paper has gone through grammatical editing.***

**Page 1, Abstract Introduction: Please define PLHIV where it first appears.**

*Thank you so much, this has been addressed.* *People Living with HIV (PLHIV), Line number 15 to 16*

**Page 1, Abstract Methods: Please define** **FGD where it first appears.**

*Thank you, this has been defined**, Focus Group Discussions (FGD) Line number 24.*

**Page 3, paragraph 1: Please define** **UPHIA where it first appears.**

*Thank you, this has been defined, Uganda Population-based HIV Impact Assessment (UPHIA) defined Line number 71 to 72.*

**Page 3, paragraph 1: By the following sentences: “For adults above 15 years the prevalence was 5.8%. HIV prevalence was estimated at 5.8% with the highest being in women at 7.2% than in men of 4.3%.” do the authors mean, “****For adults above 15 years, HIV prevalence was estimated at 5.8% with higher prevalence in women (at 7.2%) than in men (4.3%).”?**

*Thank you for the correction, this has been adjusted, line number 73 to 74*

**Page 3, paragraph 1: What is the “90% target”? Is this different from the 95% targets mentioned in the paragraph before?**

*Thank you for your observation. Yes, the UNAIDS 90% targets were revised to 95%. The 95:95:95 targets are intended to achieve 95% testing coverage, 95% treatment coverage and 95% Viral load suppression. Retention in care directly affects treatment coverage and in order to achieve the second 95% target, the retention in care should also be the same (95% retention=95% treatment coverage). Line number 83 to 84*

*“…ranging from 65-75% against the UNAIDS 95% target”*

**Page 5, paragraph 1: Should “Bufumira HCII” be “Bufumira HCIII” instead?**

***Thank you for your observation, its Bufumira HCIII. This has been corrected on line number 164***

**Page 6, paragraph 2: If possible, more information about how participants were assigned to FGD vs IDI would be helpful.**

*We appreciate your advice, this has been added, line number 176 to 213*

**Reviewer #4**: Perceptions and attitudes towards unmanned aerial vehicles (drones) use for delivery of HIV medication among fisher folk communities on the Islands of Kalangala, Uganda

PGPH-D-23-01417

Overall

**The paper addresses an important issue on stakeholder perceptions on use of medical drones to deliver ART in hard to reach islands of Kalangala District. Authors should read the paper and edit it. There are several missing words and other editorial issues that should be attended to.**

*Thank you so much, we have done a grammatical review*

Here are specific suggestions for consideration.

Abstract

1**.Write in full some abbreviated words on first mention eg PLHIV, FGDs**

*Thank you so much, this has been corrected. People Living with HIV (PLHIV) line number 15, Focus Group Discussions (FGD) line number 24*

**2. Results be consistent on use of caps. For instance, in some instances ‘medical drones’ in others Medical Drones**

*We appreciate your observation, this has been changed in the whole manuscript. For example on line number 18*

**3. Replace ‘respondents’ with participants, ‘majority’ with most….given the qualitative nature of the study.**

*Thank you for the advice. These have been replaced for example line 314.*

**Background**

**1. Authors provide a good background that links the study to the 95,95,95 global targets.**

*We thank you so much for your review.*

**2. These sentences ‘According to UPHIA 2020, the estimated prevalence of people living with HIV/AIDS was 5.5%. For adults above 15 years the prevalence was 5.8%. HIV prevalence was estimated at 5.8%’ are confusing give them a second look and edit accordingly. The HIV prevalence of 5.5% Vs 5.8% are these from different sources?**

*Overall prevalence is 5.5%. Prevalence specifically for adults above 15 years was at 5.8%. This is from the same source and has been edited. See line number 71 to 75*

**3. Recent reports indicated low retention rates for clients initiated in HIV care, ranging from 65-75% against the 90% target. Indicate the year.**

*We thank you, this has been added, line number 82*

*“Recent reports published in 2018 indicated …”*

**4. Edit sentence starting ‘Furthermore, DSD can difficult (be is missing), in is repeated. Next sentence ‘is’ seems misplaced.**

*Thank you so much for your observations, these grammatical errors have been corrected*

**5. Paragraph starting ‘ Kalangala…….18-25%; delete ‘which’**

*Thank you so much for your observations, these grammatical errors have been corrected*

**6. 24% of PLHIV being lost to follow-up add ref**

*Thank you so much for review, this has been edited, it is from reference (10)*

**7.** **Second last paragraph under background; It would be helpful to describe the level at which the drones are planned to deliver ARVs. Will the delivery point be HCs, group setting, peer or individual households? Also explain how the delivery points will be located by the drone.**

*Thank you so much for your advice.*

*“The drone will deliver drugs to peer groups under the Community Client Led ART Delivery (CCLAD). Clients will form groups of 7 people maximum, choose a group leader that picks drugs on their behalf, takes refills for their members and distributes at time and place of agreed upon. Flight routes from the health center to the delivery points will be input into the drone system. The drone will detect a QR coded piece of cloth at a delivery point and will land where the cloth is placed (precision landing)”. Line number 115 to 124*

**8. Last sentence ‘delivery’ in on (edit).**

*Thank you, this has been edited.*

**Methods**

**1.1st sentence qualitative study ‘was’ is misplaced.**

*Thank you so much, grammatical errors have been addressed.*

**2.Delete the objective from study design, its already mentioned under background.**

*We appreciate your observation, this has been deleted line number 146.*

**3.This sub study was implemented carried out (edit)**

*Thank you so much, grammatical errors have been addressed line number 149 to 150.*

**4. Study setting: edit to read as: eight settlements and surrounding**

*Thanks for your review, this has been edited, line number 160*

**5. Page 6; key opinion leaders were selected delete (s)**

*Thank you, this has been edited.*

**6. Page , under data collection; We later explained the intended use of the drones….provide the explanation that was given to participants.**

*We have added the explanation. We explained to the participants that the drones were going to deliver ART from the Health center to the Island settlements. Line number 251 to 256*

**7. Results: These are generally clearly presented**

*Thank you so much for your review*

**8. Theme 1: The community members across……(were these most? A few?). Across all results authors should show the pattern in participants views.**

*We appreciate your observation; the results have been revised.*

**9. 2.5 edit (adherence is repeated). The quote is more on missing appointments than adherence.**

*Yes, thank you. We have clarified as:*

*“2.5 “Improved adherence through improved access to medication” line number 384*

*“Almost all participants understood that if the medical drone was to deliver their medicines, then a peer would receive on their behalf and then distribute it. They believed that through drone and peer delivery, they would miss fewer appointments and thus take their ART more regularly.”*

**10. Some descriptions such as Programmers’ officer, male (could be identified). Who are these program officers and how many are they? Also, page 14, assistant fisheries Officer, male… could be identified. Add this description under methods to re-assure the readers that these officials cannot be identified.**

*Thank you so much for the observation, there are a number of program’s officers and fisheries officers, but for purposes of further anonymity, we have removed the description. For example, Line number 391*

**11. 2.7 replace majority with most.**

*Thanks, this has been corrected across the results for example line number 410*

**12. 3.3. The description is too short to stand on its own as a sub-section and paragraph. Consider expanding it or merging it with another section.**

*Thank you for the review, I have expanded on it. Line number 463*

**13. Table 2, should be brought at the start of results section, referred to in text and with few sentences highlighting key characteristics in the table.**

*Thank you for the guidance. The table has moved and some explanation has been provided. From line number 294*

**14. Discussion: This section should be strengthened. Authors should start with a brief summary of key findings and what they mean before moving on to general literature on medical drones.**

*We have added a section summarizing key findings as follows:*

*“Our findings highlight the perceived benefits and risks of medical drone use among a variety of participants in a hard-to-reach region of Uganda. We found evidence of perceived benefits around many critical elements in health service delivery including, reduced workload for health care providers, timely access to medication, reduced cost in transport, improved adherence to medical appointments. These are key factors that address important barriers in this region of the country. The perceived risks that of potential for reduced contact with health workers if medical drones efficiently deliver ART, possible increased stigma if medical drones only deliver HIV drugs and a fear of keeping the medical drone safe were mentioned. These perceived risks are understandable and important, but are certainly also not complicated to mitigate.” Line number 505*

**15. The 1st sentence, paragraph 2, starting ‘despite the growing interest…….fits in background.**

*Thank you so much, we have moved this sentence to the background section of the paper. Line number 135*

**16. Next sentence: A recently published study from Germany…..1st present your findings and what they mean then compare with other studies.**

Thank you, *we have added a brief summary of findings in the first paragraph of the discussion section****.***

**17. Page 16, edit the 1st paragraph. Acceptability of medical (drone is missing), …..which is largest (the is missing before largest). Our findings have highlighted …….then (should be than). Read and edit the paper.**

*We appreciate the correction; the paper has under gone grammatical review. Line number 528,529,531 respectively.*

**18. As mentioned, there are several interesting findings which authors have not discussed.**

*We have expounded on our discussion.*

**19. Are there any strengths of this study?**

*Yes, they have been included line number 573 to 575*

*“The strength of this study is linked to how we engaged the community at every stage of the project in order to gain key insights that answered our research question. We worked with the local leaders to support the identification and recruitment of participants into the study”.*

**Authors provided an explanation about medical drones could this have biased stakeholders?**

*Thank you so much, the explanation provided was just the intended use of the medical drones, specifically that the drones were going to be transport ART from the Health center to the island settlements, nothing more was added as seen on line number 251 to 256*

**20. Conclusion: The study provided valuable information to support project implementation……Explain briefly here or under discussion what information you mean and how helpful it was during implementation.**

*Thank you for advice, this has been added*

*Some of the valuable information that we got included; using the medical drones to deliver other medical supplies like blood so that the people receiving ART by medical drones are not stigmatized; we increased the number of island settlements receiving ART by medical drones from three (Kitoobo, Kaazi and Kusu) to six by including Buwunge, Nabumba and Nakawa as advised by the HCWs in the FGD because of the long distance; from the interviews, it was noted that apart from seeing the drones in general on television, our participants did not know about medical drones so we had to increase sensitization before implementation. Line number 583*
