# Supplementary material for: Perceptions and attitudes towards unmanned aerial vehicles (drones) use for delivery of HIV medication among fisher folk communities on the Islands of Kalangala, Uganda
Source: PLOS Glob Public Health. 2024 Aug 22;4(8):e0003468. doi: 10.1371/journal.pgph.0003468 (PMC11340983; doi:10.1371/journal.pgph.0003468)
Supplement: Attachment — Submitted filename: Reviewer report 2.docx [file pgph.0003468.s004.docx]

I congratulate the authors for a good job done. They have addressed the comments adequately. This has improved the manuscripts significantly. However, I would like them to consider the following:

Grammar editing: There are still grammatical errors.

Line 130-131. During which period did the drowning occur?

How did the authors ensure the anonymity and confidentiality of participant information? It is critical to explain to readers how you ensured it.
