# Supplementary material for: Perceptions and attitudes towards unmanned aerial vehicles (drones) use for delivery of HIV medication among fisher folk communities on the Islands of Kalangala, Uganda
Source: PLOS Glob Public Health. 2024 Aug 22;4(8):e0003468. doi: 10.1371/journal.pgph.0003468 (PMC11340983; doi:10.1371/journal.pgph.0003468)
Supplement: Attachment — Submitted filename: response to reviewer 29 May 24.docx [file pgph.0003468.s005.docx]

29 May 2024

Dear Reviewer,

Thank you for your comments and recommendations.

We have addressed the reviewer comments as follows:

1 - Grammar editing: There are still grammatical errors.

We have gone through the entire manuscript and edited for grammar.

2 - Line 130-131. During which period did the drowning occur?

Dates have been added from the references.

3 - How did the authors ensure the anonymity and confidentiality of participant information? It is critical to explain to readers how you ensured it.

A section on has been added in the ethics paragraph on exactly how confidentiality and anonymity were ensured as follows.

“Informed written consent was obtained from every participant prior to participation; and, the ability to withdraw from the study at any point was communicated. Participants were also assured of confidentiality and the anonymity of recordings; their participation and withdrawal were voluntary, and that gathered information would be used solely for the purpose of the study. Qualitative interview records and recorders have been kept under key and lock. Case Record Forms and other source documents have also been kept in locked cabinets. Access to databases have been given to authorized personnel only (members of the immediate study team) and a log of authorized personnel has been stored in the trial master file. This access and safety monitoring of data has been done by REDCAP team based at IDI. CRF and trial documents are kept in locked cabinets. No participant identifying information has been disclosed in any publication or at any conference activities arising from this study.”
